# Supplementary figures and images for: Epigenetic Landscapes Explain Partially Reprogrammed Cells and Identify Key Reprogramming Genes
Source: PLoS Comput Biol. 2014 Aug 14;10(8):e1003734. doi: 10.1371/journal.pcbi.1003734 (PMC4133049; doi:10.1371/journal.pcbi.1003734)

A. Continuous Cell Type Correlation Matrix

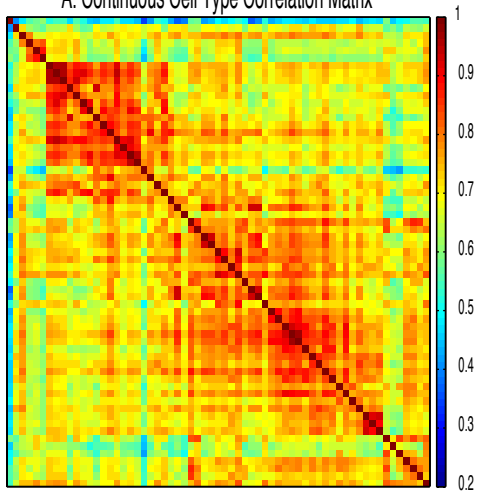

B. Binarized Cell Type Correlation Matrix

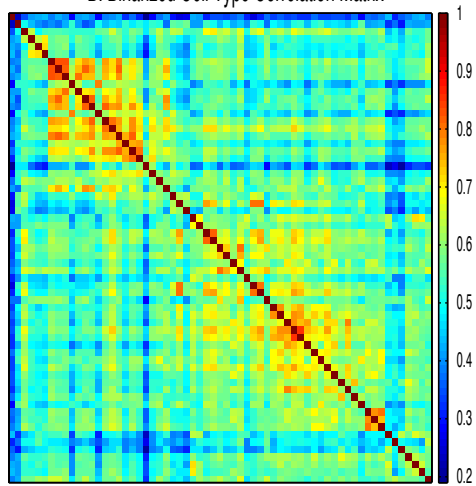

Supplement: Figure S1 — Cell fate correlation matrices. (A) Correlation matrix between cell fates for continuous data. (B) Correlation matrix for binarized data. (PDF) [file pcbi.1003734.s001.pdf]

## Random Vector Projection on Natural Cell Types

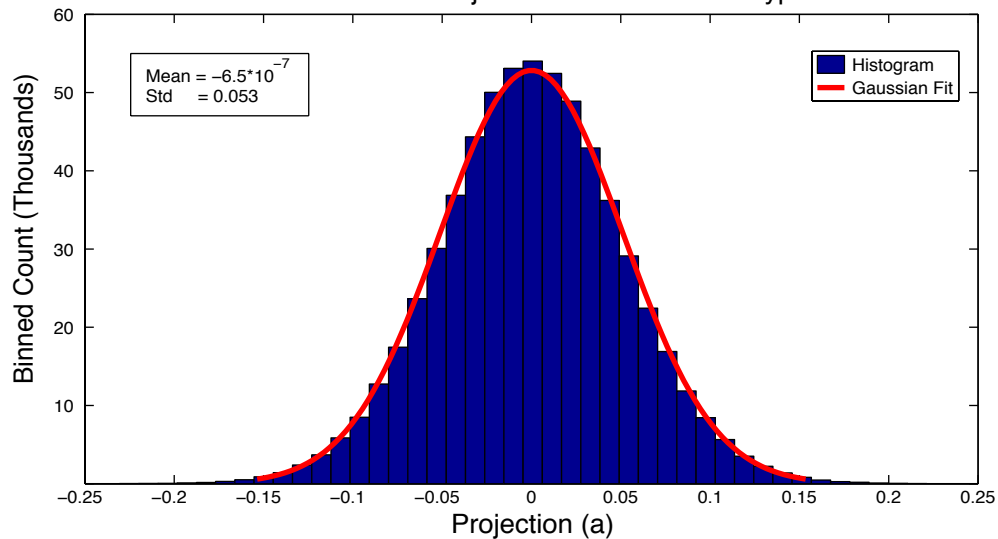

Supplement: Figure S2 — Projection of a random vector on a given cell fate. Ten thousand binarized random vectors were created in MATLAB and projected onto the cellular sub-space. The histogram shows the distribution of the projections. The red line is a Gaussian fit to the histogram. The mean is practically zero while the standard deviation is 0.053. (PDF) [file pcbi.1003734.s002.pdf]

# NSC

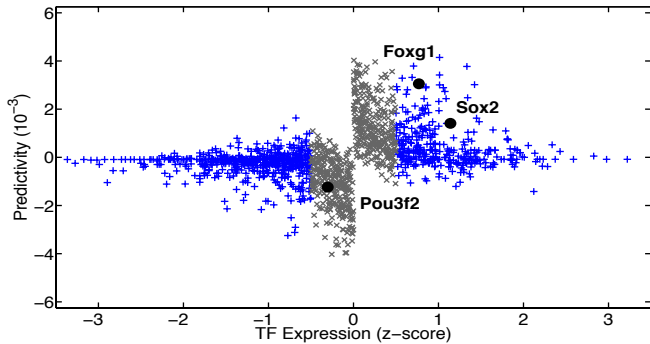

Supplement: Figure S3 — Predictivity vs expression for NSC. Same type of plot as Figure 3. Labeled TFs are part of reprogramming protocol to NPC [6]. This illustrates that Foxg1 is predictive for NSC, even though it is not for NPC. (PDF) [file pcbi.1003734.s003.pdf]
