# Supplementary material for: Epigenetic Landscapes Explain Partially Reprogrammed Cells and Identify Key Reprogramming Genes
Source: PLoS Comput Biol. 2014 Aug 14;10(8):e1003734. doi: 10.1371/journal.pcbi.1003734 (PMC4133049; doi:10.1371/journal.pcbi.1003734)
Supplement: Table S1 — Classifying top ESC reprogramming candidates. Table has top 50 embryonic stem cell (ESC) reprogramming candidates (as ranked by z-score times predictivity, ). Classification of each TF is either justified by paper citation or GO Process term. (PDF) [file pcbi.1003734.s010.pdf]

Table S1. Classifying Top ESC Reprogramming Candidates.

TF	Z Score	η (10^{-3})	Rank Z$*\eta$	Classification	Citation or GO Term
Pou5f1 (*Oct4*)	2.77	2.45	1	Reprogramming	[1]
Gm13242	1.59	3.98	2	Unknown	biological process
Nr0b1	2.44	2.59	3	Pluripotency	[2]
Nanog	2.30	2.65	4	Reprogramming	[1]
Zfp42	2.04	2.74	5	Pluripotency	[3]
Hsf2bp	1.42	3.49	6	Unknown	biological process
Esrrb	1.74	2.49	7	Reprogramming	[1]
Zscan4f	1.01	3.86	8	Reprogramming	[4]
Klf4	1.04	3.25	9	Reprogramming	[1]
Zfp459	0.83	3.98	10	Unknown	biological process
Zscan4c	0.82	3.86	11	Pluripotency	telomere lengthening
Zic3	1.17	2.65	12	Pluripotency	[5]
Zfp936	1.15	2.66	13	Unknown	biological process
Zfp229	0.76	3.84	14	Unknown	biological process
Zfp600	0.71	3.98	15	Unknown	biological process
Zfp640	1.10	2.55	16	Differentiation	skeletal system morphogenesis
Gm10324	1.09	2.55	17	Unknown	biological process
Zscan10	1.04	2.65	18	Pluripotency	[6]
Utf1	2.03	1.30	19	Reprogramming	[1]
2610305D13Rik	1.02	2.45	20	Unknown	biological process
Tfcp2l1	1.26	1.90	21	Pluripotency	[7]
Klf8	0.58	4.12	22	Differentiation	[8]
Epas1	0.70	3.18	23	Differentiation	erythrocyte differentiation
Tbx3	1.09	2.03	24	Reprogramming	[1]
Tcf15	0.89	2.37	25	Differentiation	[9]

Table has top 50 embryonic stem cell (ESC) reprogramming candidates (as ranked by z-score times predictivity, η_i^μ). Classification of each TF is either justified by paper citation or GO Process term. Reprogramming TFs are in a pre-existing reprogramming protocol, pluripotency TFs help maintain the ESC state but are non-essential for reprogramming, differentiation TFs are expressed in ESC but help induce cell fate change *in vivo*, and unknown TFs have no known function.

Table S1 Continued. Classifying Top ESC Reprogramming Candidates.

TF	Z Score	η (10^{-3})	Rank Z$*\eta$	Classification	Citation or GO Term
Tcfl5	0.82	2.56	26	Unknown	regulation of transcription
Sall4	1.72	1.17	27	Reprogramming	[1]
Zfp553	0.87	2.22	28	Unknown	regulation of transcription
Sox2	1.96	0.97	29	Reprogramming	[1]
Grhl3	0.61	2.75	30	Differentiation	ectoderm development
Zbtb10	0.75	2.22	31	Unknown	negative regulation of transcription
Mycn	1.90	0.85	32	Differentiation	lung development
Sap30	0.93	1.72	33	Differentiation	skeletal muscle cell differentiation
Zbtb8a	0.83	1.88	34	Unknown	regulation of transcription
Klf5	1.23	1.25	35	Differentiation	skeletal muscle cell differentiation
Sall1	1.30	1.18	36	Differentiation	neural tube development
AA987161	0.60	2.36	37	Unknown	biological process
Klf9	0.70	1.96	38	Differentiation	embryo implantation
Myc	0.73	1.86	39	Reprogramming	[1]
Rarg	0.87	1.54	40	Differentiation	bone morphogenesis
Tead2	1.03	1.15	41	Differentiation	lateral mesoderm development
Dnmt3b	1.33	0.88	42	Pluripotency	genetic imprinting
Nr5a2	0.67	1.75	43	Reprogramming	[1]
Nr1d2	0.74	1.53	44	Differentiation	regulation of skeletal muscle cell differentiation
Cbx7	1.14	0.99	45	Differentiation	chromatin modification
Bnip3	1.40	0.77	46	Differentiation	brown fat cell differentiation
Rbpms	1.63	0.64	47	Unknown	transcription, DNA-templated
Zfp7	0.91	1.15	48	Unknown	regulation of transcription, DNA-templated
Lin28a	0.78	1.31	49	Reprogramming	[1]
Zfp423	0.55	1.79	50	Differentiation	cell differentiation

---

[1] González F, Boué S, Belmonte JCI (2011) Methods for making induced pluripotent stem cells: reprogramming àla carte. Nat Rev Genet 12: 231-242.

[2] Khalfallah O, Rouleau M, Barbry P, Bardoni B, Lalli E (2009) Dax-1 knockdown in mouse embryonic stem cells induces loss of pluripotency and multilineage differentiation. STEM CELLS 27: 1529–1537.

[3] Takahashi K, Yamanaka S (2006) Induction of pluripotent stem cells from mouse embryonic and adult fibroblast cultures by defined factors. Cell 126: 663-676.

[4] Jiang J, Lv W, Ye X, Wang L, Zhang M, et al. (2013) Zscan4 promotes genomic stability during reprogramming and dramatically improves the quality of ips cells as demonstrated by tetraploid complementation. Cell Res 23: 92–106.

[5] Lim LS, Loh YH, Zhang W, Li Y, Chen X, et al. (2007) Zic3 is required for maintenance of pluripotency in embryonic stem cells. Molecular Biology of the Cell 18: 1348-1358.

[6] Yu Hb, Kunarso G, Hong FH, Stanton LW (2009) Zfp206, oct4, and sox2 are integrated components of a transcriptional regulatory network in embryonic stem cells. Journal of Biological Chemistry 284: 31327-31335.

[7] Ye S, Li P, Tong C, Ying QL (2013) Embryonic stem cell self-renewal pathways converge on the transcription factor tfcp2l1. EMBO J 32: 2548–2560.

[8] Lee H, Kim HJ, Lee YJ, Lee MY, Choi H, et al. (2012) Krüppel-like factor klf8 plays a critical role in adipocyte differentiation. PLoS ONE 7: e52474 EP -.

[9] Davies OR, Lin CY, Radzisheuskaya A, Zhou X, Taube J, et al. (2013) Tcf15 primes pluripotent cells for differentiation. Cell Reports 3: 472–484.

[10] Young RA (2011) Control of the embryonic stem cell state. Cell 144: 940-954.
