# Supplementary material for: Epigenetic Landscapes Explain Partially Reprogrammed Cells and Identify Key Reprogramming Genes
Source: PLoS Comput Biol. 2014 Aug 14;10(8):e1003734. doi: 10.1371/journal.pcbi.1003734 (PMC4133049; doi:10.1371/journal.pcbi.1003734)
Supplement: Table S2 — Examining Yamanaka factors in detail. Here we reexamine the Yamanaka transcription factors (TFs) in light of our model. (PDF) [file pcbi.1003734.s011.pdf]

Table S2. Examining Yamanaka Factors in Detail

TF	(A) Exp.	(B) Diff. Exp.	(C) Specificity	(D) Predictivity	(E) Exp*Pred
Pou5f1 (*Oct4*)	2	1	1.6%	70	1
Sox2	22	11	0.0%	201	29
Klf4	122	124	22.2%	28	9
Myc	213	1183	66.7%	106	39

Here we reexamine the Yamanaka transcription factors (TFs) in light of our model. When the Yamanaka results were first published, *Klf4* and *Myc* were counterintuitive factors[1]. *Myc* was quickly shown to enhance the efficiency of reprogramming but was dispensable[2]. *Klf4* remained a surprise, but this table demonstrates the power of predictivity by establishing the importance of *Klf4*. The columns (A),(B), and (C) are data about TFs available to Yamanaka, while (D) and (E) are data from our model. Unless otherwise stated, the numbers represent rank order (out of 1337) relative to the other TFs. To understand the importance of rank order, the original Yamanaka experiment used 24 TFs while most later studies test around 10 TFs at once. (A) Exp. is TF expression rank in embryonic stem cells (ESC). (B) Diff. Exp. is the differential expression rank between ESC and mouse embryonic fibroblasts (MEF), the starting cell fates in the Yamanaka protocol. (C) Specificity is the percentage of cell fates (out of our 63) which have expression at the same or higher level as the ESC. (D) Predictivity is the novel measure of TF importance generated by our model. (E) Exp*Pred is the rank of the product of expression and predictivity of highly expressed TFs and is an attempt to find a single quantity signifying reprogramming potential. The data available to Yamanaka illustrates that *Pou5f1* (*Oct4*) and *Sox2* were natural choices. *Myc* is an oncogene that enhances proliferation but was found to be non-essential for reprogramming[2], so we will ignore it. The power of predictivity is illustrated by examining *Klf4* which is not highly expressed (A), differentially expressed (B), or specific (C). However, it is very predictive of ESC (D) and is a top choice when examining Exp*Pred (E). Note that *Klf4* illustrates that predictivity is not exactly the same as specificity. While *Klf4* is expressed in many cell fates, since predictivity takes into account correlations between cell fate expression patterns, predictivity can filter out the uncorrelated expression pattern and highlight the importance of *Klf4* for ESC.

---

[1] Jaenisch R (2012) Nuclear cloning and direct reprogramming: The long and the short path to stockholm. Cell Stem Cell 11: 744–747.

[2] Wernig M, Meissner A, Cassady JP, Jaenisch R (2008) c-myc is dispensable for direct reprogramming of mouse fibroblasts. Cell Stem Cell 2: 10-12.
