# Supplementary material for: Epigenetic Landscapes Explain Partially Reprogrammed Cells and Identify Key Reprogramming Genes
Source: PLoS Comput Biol. 2014 Aug 14;10(8):e1003734. doi: 10.1371/journal.pcbi.1003734 (PMC4133049; doi:10.1371/journal.pcbi.1003734)
Supplement: Text S1 — Attractor neural networks: Additional details. This supplementary text provides extended background details on Hopfield attractor neural networks but presents no new research findings. The sections are: (A) Discrete, Standard Hopfield. (B) Continuous, Standard Hopfield. (C) Continuous Gene Expression. (D) Discrete as Limit of Continuous. (E) Discrete, Projection Method. (PDF) [file pcbi.1003734.s012.pdf]

Supplementary Information: Epigenetic landscapes explain partially reprogrammed cells and identify key reprogramming gene

Alex H. Lang,[1,2] Hu Li,[3,4] James J. Collins,[3,4,5,6] and Pankaj Mehta[1,2]

[1]Physics Department, Boston University, Boston, MA, USA
[2]Center for Regenerative Medicine, Boston University, Boston, MA, USA
[3]Department of Biomedical Engineering, Boston University, Boston, MA, USA
[4]Wyss Institute for Biologically Inspired Engineering, Harvard University, Boston, MA, USA
[5]Howard Hughes Medical Institute, Boston, MA, USA
[6]Center for BioDynamics, Boston University, Boston, MA, USA

Contents

I. Attractor Neural Networks: Additional Details 1
A. Discrete, Standard Hopfield 1
B. Continuous, Standard Hopfield 4
C. Continuous Gene Expression 4
D. Discrete as Limit of Continuous 5
E. Discrete, Projection Method 5

References 6

I. ATTRACTOR NEURAL NETWORKS: ADDITIONAL DETAILS

This supplementary text gives a brief introduction to Hopfield neural networks[1,2] and how they can be adapted to study epigenetic landscapes. We begin by reviewing the basic principles underlying the original Hopfield neural network. We then show how to generalize this to continuous spins[3] as well as discrete spins with correlated cell fates[4] (projection method). For an in-depth introduction to neural networks, please see the beautiful book by Amit[5].

A. Discrete, Standard Hopfield

There are N genes and each gene i is either on or off, with the output denoted by $S_i = \pm 1$. Alternatively, we could use the variables $\widetilde{S} = \frac{1}{2}(S+1) = 1, 0$ with the corresponding substitutions in all equations below.

The input to a given gene i is denoted by the local field

$$h_i = \sum_{j \neq i}^{N} J_{ij} S_j + B_i \tag{1}$$

where J_{ij} is the interaction between gene i and gene j and B_i is the external (i.e interaction independent) bias of gene i. Both J_{ij} and B_i are assumed to be independent of S_i.

The landscape H is given by

$$H = -\frac{1}{2} \sum_{i=1}^{N} \sum_{j \neq i}^{N} S_i J_{ij} S_j - \sum_{i=1}^{N} B_i S_i \tag{2}$$

$$= -\frac{N}{2} \sum_{\mu=1}^{p} (m^\mu)^2 - N \sum_{\mu=1}^{p} b^\mu m^\mu \tag{3}$$

where in equation 3 we have introduce the order parameter for the overlap (dot product or "magnetization") of a spin configuration with a given cell fate μ as m^μ and also introduced the cell fate bias b^μ. The overlap is defined in terms

of the cell fate vectors ξ_i^μ as:

$$m^\mu = \frac{1}{N}\sum_{i=1}^{N} \xi_i^\mu S_i \tag{4}$$

To prove that H is a Lypanov function (i.e. has stable equilibrium states and follows the standard definition of an "energy"), it is necessary to show that H is a decreasing function and bounded below. To do so, consider flipping a single S_i. The resulting change in H is

$$\Delta H = -\frac{1}{2}\left[\sum_{j\neq i}^{N} J_{ij} S_j + \sum_{j\neq i}^{N} S_j J_{ji} + B_i\right]\Delta S_i \tag{5}$$

When we have symmetric interactions, $J_{ij} = J_{ji}$, this simplifies to

$$\Delta H = -\left[\sum_{j\neq i}^{N} J_{ij} S_j + B_i\right]\Delta S_i = -h_i \Delta S_i \tag{6}$$

To determine the sign of ΔH we need the relation between h_i and ΔS_i. For deterministic (stochastic) dynamics, as long as ΔS_i and h_i are always (usually) the same sign, we always (usually) have $\Delta H < 0$. Therefore, any set of dynamics that stochastically matches the sign of ΔS_i and h_i will lead to H being a Lypanov function. This implies that any choice of dynamics leads to the same stable fixed points, but may give rise to different trajectories, limit cycles, and sizes of basins of attraction for fixed points, see Amit[5] section 2.2 and 3.5 for a detailed analysis. Therefore, in this paper we focus on predictions that are independent of the exact dynamics. This is equivalent to thinking about the stationary properties of the model.

We will follow the standard convention for neural networks and physics and implement Glauber dynamics which is an asynchronous, stochastic update rule. In this update scheme, at each time step, one gene is selected at random and probabilistically updated according to its local field

$$P[S_i(t+1)] = \frac{e^{\beta h_i(t) S_i(t+1)}}{e^{\beta h_i(t)} + e^{-\beta h_i(t)}} \tag{7}$$

with h_i defined above (or equivalently $h_i = -\frac{\partial H}{\partial S_i}$) and t time measured in discrete updates. Also, $\beta = 1/T$ is the inverse temperature and characterizes the slope of the sigmoid function. When $\beta \to \infty$, the sigmoid approaches a deterministic step function, while when $\beta \to 0$ each state is equally likely.

Now we need to specify the gene interaction J_{ij} and establish the global minima of the system. There are p cell fates and the state of gene i in cell fate μ is given by ξ_i^μ. The gene interaction is a correlation based interaction and in the standard Hopfield neural network it is defined as

$$J_{ij} = \frac{1}{N}\sum_{\mu=1}^{p} \xi_i^\mu \xi_j^\mu \tag{8}$$

In the standard Hopfield network, the cell fates have two assumptions. First, each cell fate is assumed to on average be unbiased (i.e. equal number of positive and negative spins)

$$\frac{1}{N}\sum_{i=1}^{N} \xi_i^\mu \approx 0 \tag{9}$$

and second every pair of cell fates is approximately orthogonal

$$\frac{1}{N}\sum_{i=1}^{N} \xi_i^\mu \xi_i^\nu \approx \mathcal{O}\left(\frac{1}{\sqrt{N}}\right) \tag{10}$$

These two assumptions can be relaxed in extensions of the standard Hopfield neural network, see later sections for one example (the projection method) that can incorporate correlated cell fates.

Now we can prove that each cell fate is a global minima of the landscape. For no external fields, the landscape can be written as:

$$H = -\frac{1}{2}\sum_{i=1}^{N}\sum_{j\neq i}^{N} S_i J_{ij} S_j = -\frac{N}{2}\sum_{\mu=1}^{p}\left(\frac{1}{N}\sum_{i=1}^{N}\xi_i^\mu S_i\right)^2 + \frac{1}{2N}\sum_{i=1}^{N}\sum_{\mu=1}^{p} S_i\xi_i^\mu\xi_i^\mu S_i \tag{11}$$

This can be rewritten in terms of the overlap as:

$$H = -\frac{N}{2}\mathbf{m}^2 + \frac{1}{2}p \tag{12}$$

Then as long as N is large compared to p, whenever we are in a given cell fate the energy is $H = -N/2$ and this is the lowest bound since $\mathbf{m}^2 \leq 1$. We have shown that for $p \ll N$, H is a decreasing, bounded function and hence is a Lypanov function. When p and N are both large, a full replica calculation shows that H remains a Lypanov function[6].

While we have established that the landscape is a Lypanov function, we also need to examine the dynamical stability of the cell fates and the existence of spurious attractors. In the absence of stochastic update noise ($\beta \to \infty$), we can examine the signal-to-noise ratio of the cell fates. If a state is dynamically stable, one needs $S_i h_i > 0$. When the state is in a given cell fate (without loss of generality assume cell fate 1), we have that

$$\xi_1^1 h_1 = \frac{1}{N}\sum_{j\neq i}^{N}\sum_{\mu}^{p}\xi_1^1\xi_1^\mu\xi_j^\mu\xi_j^1 \tag{13}$$

which can be broken into a signal term (first term) and noise term (second term) as follows:

$$\xi_1^1 h_1 = \frac{N-1}{N} + \frac{1}{N}\sum_{j\neq i}^{N}\sum_{\mu\neq 1}^{N}\xi_1^1\xi_1^\mu\xi_j^\mu\xi_j^1 \tag{14}$$

For large N, the signal term approaches 1. We can evaluate the noise term by recognizing that it is an unbiased sum of $(N-1)(p-1) \approx Np$ random steps, and therefore has mean 0 and standard deviation $\sqrt{pN}$, giving us

$$\xi_1^1 h_1 = 1 + \mathcal{O}\left(\sqrt{\frac{p}{N}}\right) \tag{15}$$

Therefore as long as N is much larger than p, every cell fate is a fixed point. This rough signal-to-noise argument can be made more rigorous by a spin-glass replica calculation[6] which finds that cell fates are stable (in the case $\beta \to \infty$) as long as the ratio of p/N is less than 0.138.

Here is an intuitive argument of why the landscape must be rugged, which implies the scaling of stable states with N. From looking at small systems, a naive guess would be that the number of stable states should scale with the size of the state space 2^N. This scaling could be achieved if each minima occurred when a single TF state is turned on while all the other TFs are off. However, this implies that each minima is only marginally stable; any spin flip will move the state out of the minima. In order to have a basin of attraction, more TFs are needed to determine the minima. A simple error correction or redundancy could be implemented by using r redundant TFs, but this would require exponentially more states r^N. Instead, stable states could be determined by overlapping sets of TFs, as in the Hopfield neural network. This form of error-correction leads to frustration and Gaussian noise between the stable states, hence the scaling of stable states with N and not 2^N.

An unavoidable consequence of the non-linearity (ruggedness) of the Hopfield network is that in addition to the desired attractors (the input cell fates), there are additional spurious, metastable, attractors. There are a variety of spurious attractors, but the most common are symmetric mixtures of odd states[2], for example without loss of generality we can make a spurious state with the first three cell types, $S_{spur} = \text{major}\,(\xi_1 + \xi_2 + \xi_3)$, where major stands for majority vote (equivalently the sign function) at each spin. The most common spurious attractor are symmetric mixtures of 3 states (as in the example above). A signal-to-noise analysis can also be done to establish that these spurious attractors are stable attractors, but with a smaller basin of attraction than the input cell fates (see Amit 4.3 for details[5]).

B. Continuous, Standard Hopfield

The previous section describes the basic ideas of Hopfield neural networks. Here, we show how discrete Hopfield neural networks can be considered a limiting case of continuous differential equations of gene expression. We start by defining continuous spins, σ_i, that can take on real number between -1 and 1. For continuous dynamics, we must modify the dynamics of the corresponding local field. In particular, if the local field decays in time with a time constant τ_i we have

$$\frac{dh_i}{dt} = \sum_{j\neq i}^{N} J_{ij}\sigma_j + B_i - \tau_i^{-1} h_i \tag{16}$$

where the J_{ij} are the same as in the discrete case and the spin σ_i is related to the local field by some monotonic function $\sigma_i = g_i\left[h_i\right]$.

Now the landscape is given by

$$H = -\frac{1}{2}\sum_{i=1}^{N}\sum_{j\neq i}^{N}\sigma_i J_{ij}\sigma_j - \sum_{i=1}^{N} B_i\sigma_i + \sum_{i=1}^{N}\tau_i^{-1}\int_{-1}^{\sigma_i} g_i^{-1}\left[\sigma\right] d\sigma \tag{17}$$

where the first two terms are the same as in the discrete case while the third is the new term for continuous only. Taking derivatives with respect to time gives us

$$\frac{dH}{dt} = -\sum_{i=1}^{N}\frac{d\sigma_i}{dt}\left(\sum_{j\neq i}^{N} J_{ij}\sigma_j + B_i - \tau_i^{-1}h_i\right) = -\sum_{i=1}^{N}\frac{d\sigma_i}{dt}\frac{dh_i}{dt} \tag{18}$$

Then since $h_i = g_i^{-1}\left[\sigma_i\right]$, we can relate the derivative of h_i to the derivative σ_i. Then using the fact that g_i is monotonically increasing we can show that the change in H is always negative:

$$\frac{dH}{dt} = -\sum_{i}^{N} g_i^{-1}\left[\sigma_i\right]\left(\frac{d\sigma_i}{dt}\right)^2 \leq 0 \tag{19}$$

The decrease in H along with the fact that H is bounded below, shows that we have a Lypanuv function. It is easy to see that every discrete stable point is also a stable point in the continuous model; however, the continuous Hopfield neural networks can have additional stable points.

C. Continuous Gene Expression

A popular approach to model gene interactions is based on the genetic toggle switch[7] and represents gene interactions by a Hill function. For now, we will use the general variable $\widetilde{\sigma} \in [\sigma_{min}, \sigma_{max}]$.

In the most general case, we have that

$$\widetilde{\sigma}_i = \text{sign}(h_i)\frac{a_i|h_i|^{n_i}}{k_i^{n_i} + |h_i|^{n_i}} + b_i \tag{20}$$

where the input h_i is in the range $[-\infty, \infty]$ and the output σ_i is in the range $[-a_i + b_i, a_i + b_i]$.

If we rescale every gene by its dynamic range and center the Hill function at zero, we get that $\widetilde{\sigma} = \sigma \in [-1, 1]$ and

$$\sigma_i = \text{sign}(h_i)\frac{|h_i|^{n_i}}{k_i^{n_i} + |h_i|^{n_i}} \tag{21}$$

Using the function above for $\sigma_i = g_i\left[h_i\right]$ allows one to relate continuous Hopfield neural networks to gene expression using Hill coefficients.

D. Discrete as Limit of Continuous

How can we relate the continuous model of gene expression to the previous discrete model? There are two limits. First, if we take the discrete time limit with the update time much greater than the input memory, we get

$$h_i(t+1) = \sum_{j\neq i}^{N} J_{ij} S_j(t) + B_i \tag{22}$$

Second, in the genetic toggle switch language, when the cooperativity is large $n \gg 1$, then $S_i \to \pm 1$. This gives us a deterministic, discrete model of gene expression. If we introduce stochasticity through Glauber dynamics, we completely recover the discrete Ising model of gene expression.

E. Discrete, Projection Method

The standard Hopfield attractor neural network assumes that the "memories" (cell fates) have nearly no correlations amongst themselves. However, cell fates are highly correlated (see Figure S1). Therefore, instead of the standard Hopfield attractor neural networks, we will implement the projection method neural networks[4].

The correlation between cell fate μ and ν is given by

$$A^{\mu\nu} = \frac{1}{N}\sum_{i=1}^{N} \xi_i^{\mu}\xi_i^{\nu} \tag{23}$$

Now the inferred correlation-based, TF interaction matrix is

$$J_{ij} = \frac{1}{N}\sum_{\mu=1}^{p}\sum_{\nu=1}^{p} \xi_i^{\mu}(A^{-1})^{\mu\nu}\xi_j^{\nu} \tag{24}$$

Then the landscape can be rewritten as

$$H = -\frac{1}{2}\sum_{i=1}^{N}\sum_{j\neq i}^{N} S_i J_{ij} S_j = -\frac{1}{2N}\sum_{i=1}^{N}\sum_{j\neq i}^{N}\sum_{\mu=1}^{p}\sum_{\nu=1}^{p} S_i \xi_i^{\mu}(A^{-1})^{\mu\nu}\xi_j^{\nu} S_j \tag{25}$$

$$= -\frac{N}{2}\sum_{\mu=1}^{p} m^{\mu} a^{\mu} \tag{26}$$

where in equation 26 we have introduced the projection order parameter a^{μ} which is the orthogonal projection of a spin vector onto the subspace spanned by the stable cell fates

$$a^{\mu} = \sum_{\nu=1}^{p}(A^{-1})^{\mu\nu} m^{\nu} = \sum_{\nu=1}^{p}\sum_{i=1}^{N}(A^{-1})^{\mu\nu}\xi_i^{\nu} S_i \tag{27}$$

A simple geometric picture illustrates that H makes each cell fate a global minimum of the landscape. An arbitrary vector can be rewritten in terms of its projection in the cell fate subspace and its orthogonal component δS_i,

$$S_i = \sum_{\mu=1}^{p} a^{\mu}\xi_i^{\mu} + \delta S_i \tag{28}$$

Then, the distance of an arbitrary vector $\mathbf{S}$ to the cell fate subspace is given by Δ,

$$\Delta = \left(\sum_{i=1}^{N}(\delta S_i)^2\right)^{1/2} \tag{29}$$

which can be rewritten as

$$\frac{\Delta^2}{N} = 1 - \sum_{\mu=1}^{p} a^{\mu} m^{\mu} \tag{30}$$

This allows us to rewrite the stabilizing term of the landscape as

$$H = -\frac{N}{2} + \frac{1}{2}\Delta^2 \tag{31}$$

This provides a very clear interpretation of the landscape as the global distance of an arbitrary vector $\mathbf{S}$ to the natural cell fate subspace[4].

Again, let's examine the signal-to-noise of cell fates in the absence of stochastic update noise. If a state is dynamically stable, one needs $S_i h_i > 0$. When the state is a given cell fate (without loss of generality assume cell fate 1), we have that

$$\xi_1^1 h_1 = \frac{1}{N}\sum_{j\neq i}^{N}\sum_{\mu=1}^{p} \xi_1^1 \xi_1^\mu \left(A^{-1}\right)^{\mu\nu} \xi_j^\nu \xi_j^1 \tag{32}$$

$$= \sum_{\mu=1}^{p} \xi_1^1 \xi_1^\mu \left(A^{-1}\right)^{\mu\nu} A^{\nu 1} = 1 \tag{33}$$

Therefore, the stability of cell fate 1 has no noise interference from the other cell fates, and we have that cell fates are stable up to $p/N = 1$.

1 Hopfield JJ (1982) Neural networks and physical systems with emergent collective computational abilities. Proc. Natl. Acad. Sci. U.S.A. 79: 2554-2558.

2 Amit DJ, Gutfreund H, Sompolinsky H (1985) Spin-glass models of neural networks. Phys. Rev. A 32: 1007-1018.

3 Hopfield JJ (1984) Neurons with graded response have collective computational properties like those of two-state neurons. Proc. Natl. Acad. Sci. U.S.A. 81: 3088-3092.

4 Kanter I, Sompolinsky H (1987) Associative recall of memory without errors. Phys. Rev. A 35: 380-392.

5 Amit D (1992) Modeling Brain Function: The World of Attractor Neural Networks. Cambridge: Cambridge Univ. Press.

6 Amit DJ, Gutfreund H, Sompolinsky H (1985) Storing infinite numbers of patterns in a spin-glass model of neural networks. Phys. Rev. Lett. 55.

7 Gardner TS, Cantor CR, Collins JJ (2000) Construction of a genetic toggle switch in escherichia coli. Nature 403: 339–342.
